# Supramolecular assemblies of rifampicin and cationic bilayers: preparation, characterization and micobactericidal activity

**DOI:** 10.1186/1472-6750-11-40

**Published:** 2011-04-15

**Authors:** Lilian Barbassa, Elsa M Mamizuka, Ana M Carmona-Ribeiro

**Affiliations:** 1 Biocolloids Lab, Departamento de Bioquímica, Instituto de Química, Universidade de São Paulo, CP 26077, CEP 05513-970, São Paulo SP, Brazil; 2 Departmento de Análises Clínicas e Toxicológicas, Faculdade de Ciências Farmacêuticas, Universidade de São Paulo, CEP 05508-900, São Paulo/SP, Brazil

## Abstract

**Background:**

Cationic bilayers based on the inexpensive synthetic lipid dioctadecyldimethylammonium bromide (DODAB) have been useful as carriers for drug delivery, immunoadjuvants for vaccines and active antimicrobial agents.

**Methods:**

Rifampicin (RIF) or isoniazid (ISO) interacted with DODAB bilayer fragments (BF) or large vesicles (LV). Dispersions were evaluated by dynamic light-scattering for zeta-average diameter (Dz) and zeta-potential (ζ) analysis; dialysis for determination of drug entrapment efficiency; plating and CFU counting for determination of cell viability of *Mycobacterium smegmatis *or *tuberculosis*, minimal bactericidal concentration (MBC) and synergism index for DODAB/drug combinations.

**Results:**

DODAB alone killed micobacteria over a range of micromolar concentrations. RIF aggregates in water solution were solubilised by DODAB BF. RIF was incorporated in DODAB bilayers at high percentiles in contrast to the leaky behavior of ISO. Combination DODAB/RIF yielded MBCs of 2/2 and 4/0.007 μg/mL against *Mycobacterium smegmatis *or *Mycobacterium tuberculosis*, respectively. Synergism indexes equal to 0.5 or 1.0, indicated synergism against the former and independent action, against the latter species.

**Conclusions:**

*In vitro*, DODAB acted effectively both as micobactericidal agent and carrier for rifampicin. The novel assemblies at reduced doses may become valuable against tuberculosis.

## Background

Tuberculosis (TB) is a curable infectious disease affecting mostly the lungs which is caused by *Mycobacterium tuberculosis*. In 2008, an increase of 9.3 million novel TB cases was reported, since the slow reduction in incidence rates per capita continues to be surpassed by increasing world population [1]. Control of TB remains a serious challenge for public health due to the emergence of mutated strains presenting resistance to at least the two major anti-tuberculosis drugs, isoniazid and rifampicin [2]. Treatment of multidrug resistant TB requires prolonged and expensive chemotherapy using highly toxic, second-line drugs [2]. Eventually, resistance to these second line drugs develops and the disease becomes untreatable [2]. Clearly, there is an urgent need to improve treatment by either enhancing the application of existing agents or introducing new drugs. Thus, it is important to develop not only new drug candidates and drug targets but also delivery systems.

Cationic bilayers based on dioctadecyldimethylammonium bromide (DODAB) have been useful as carriers in drug delivery [3], immunoadjuvants for vaccines [4,5] and as antimicrobial agents due to their quaternary ammonium moiety [6]. In this work, the ability of such bilayers to incorporate and deliver antitubercular drugs such as rifampicin (RIF) and isoniazid (ISO) *in vitro *was investigated and assemblies tested against two *Mycobacterium *species. The results evidenced the micobactericidal activity *in vitro *of DODAB alone or in combination with rifampicin.

## Methods

### Materials

**Figure 1:**
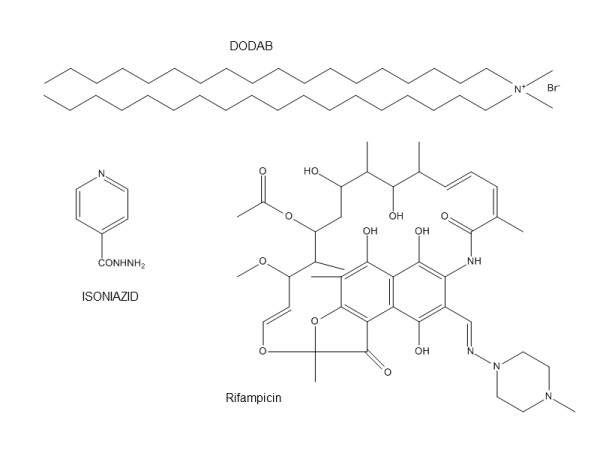
**Chemical structures of DODAB cationic lipid and antitubercular drugs RIF and ISO**. The chemical structures of isoniazid (ISO), rifampicin (RIF) and dioctadecildimetilamonium bromide (DODAB).

DODAB 99.9% pure, dipalmitoylphosphatidylcholine (DPPC) and asolecitin (ASO) were obtained from Sigma (USA) and used as such without further purification. RIF and ISO were obtained from Sigma-Aldrich, USA. Anhydrous D-glucose was from Merck (Darmstadt, Germany) and water was Milli-Q quality. Chemical structures of DODAB, RIF and ISO were shown in Figure 1.

### Preparation of DODAB dispersions

Lipids were dispersed in water or 0.264 M D- glucose solution. DODAB bilayer fragments (BF) were prepared by sonication with tip above the gel-to-liquid-crystalline bilayer phase transition temperature [7,8]. This procedure dispersed the amphiphile powder in water using a high-energy input, which not only produced bilayer vesicles but also disrupted these vesicles, thereby generating open BF. DODAB was alternatively dispersed by vortexing at 60°C to obtain LV. DODAB analytical concentration was determined by bromide microtitration [9].

### Determination of size distribution, zeta-average diameter and zeta-potential for dispersions

The size distributions for DODAB, RIF and DODAB/RIF were determined by means of a zeta-potential analyser (ZetaPlus-Brookhaven Instruments Corporation, Holtsville, NY, USA) equipped with a 570 nm laser and dynamic light scattering at 90° for particle sizing [10]. The zeta-average diameter (Dz) referred to in this work should be understood as the mean hydrodynamic diameter. Zeta-potentials (ζ) were determined from the electrophoretic mobility μ and Smoluchowski's equation, ζ = μη/ε, where η and ε are medium viscosity and dielectric constant, respectively. All D*z *and ζ were obtained at 25°C, 1 h after mixing.

### Determination of RIF or ISO optical spectra in different media

RIF or ISO optical spectra were determined using a Hitachi 2000 spectrophotometer in the double-beam mode against a blank of lipid dispersions and solvent (without drug). RIF and ISO stock solutions were prepared at 1 mg/mL in dimethylsulfoxide (DMSO) and water, respectively. RIF spectra were obtained by adding RIF stock solution to water, DMSO, DODAB BF or DODAB LV. ISO spectra were obtained by adding the stock solution to water or DODAB LV dispersions. All optical spectra were obtained at 25° C just after mixing. Wavelengths of maximal absorption for each drug in different media plus drug molar absorptivity were determined from analysis of optical spectra.

### Determination of entrapment efficiency for RIF in DODAB dispersions

DODAB LV or BF were obtained in a 0.1 mM RIF and 0.264 M D-glucose solution. Aliquots of 2 mL of the RIF solution (control) and DODAB BF or LV (at 2 mM DODAB) prepared in this RIF solution were dialyzed against (4X) 250 mL of 0.264 M D-glucose solution for 24 h. Thereafter, aliquots of RIF solution, DODAB LV/RIF or DODAB BF/RIF before and after dialysis were added of ethanol (50%) in order to solubilize the DODAB bilayers.RIF concentration was determined spectrophotometrically from absorbance at 334 nm. Drug incorporation percentile (% Inc) was given by equation (1):(1)

Where A_1 _and A_2 _are absorbances of RIF/DODAB LV or RIF/DODAB BF before and after dialysis, respectively; A _1c _and A _2c _are absorbances of RIF solution before and after dialysis. Since % Inc depends on DODAB concentration, % Inc was normalized to DODAB concentration yielding entrapment efficiency (ENT) from equation (2):(2)

ENT in L/mol depends only on physical characteristics of the liposomal dispersion such as size and lamelarity but does not depend on lipid concentration.

### Determination of ISO entrapment efficiency in DODAB, asolecithin (ASO) or DODAB/DPPC 1:1 dispersions

DODAB LV were prepared in a 0.1 mM ISO solution at 2 mM DODAB. Large DODAB/DPPC vesicles were prepared by spreading and evaporating a chloroform solution of lipids on the bottom of a glass tube [11]. Briefly, films of DODAB/DPPC at 2 mM total lipid were prepared from stock chloroform solutions of the lipids by evaporating the solvent under N_2 _flux. In order to remove the residual solvent, the films remained under vacuum overnight at room temperature. Vesicles were prepared by hydrating the films with 0.1 mM ISO water solution at 60° C and vortexing dispersions until they became homogeneous. Asolecitin lipids at 2 mM total lipid were dispersed by vortexing at room temperature to obtain multilamellar vesicles (MLV) in the same 0.1 mM ISO solution [12]. The dialysis procedure employed to determine ISO entrapment in the three different lipid dispersions was similar to the one described for RIF in the previous item. Dialysis was carried out against water for 12 h. After dialysis, the ISO contents inside each bag were evaluated at 260 nm after liposomes lysis either by adding ethanol (50%) or Triton X-100 (1%). % Inc and ENT were calculated as described in the previous section.

### Microrganisms and culture conditions

*M. smegmatis *(ATCC mc2155) and *M. tuberculosis *(ATCC 27294) were obtained from Mycobacterial Laboratory culture collection of the Universidade de São Paulo. *M. smegmatis *was cultured freshly on Muller Hinton Agar (MHA) plates for 72 h at 30°C. Thereafter, one or two colonies were transferred to 10 mL Middlebrook 7H9 broth and incubated in a shaker (150 rpm/30°C/48 h). *M. tuberculosis *was cultured freshly on Lowenstein- Jensen (BBL™ - Becton Dickinson Microbiology System, Sparks, MD, USA) agar for 28 days at 37° C. Thereafter, one or two colonies were transferred to 10 mL of Middlebrook 7H9 culture medium supplemented with 10% oleic acid albumin dextrose complex (OADC) and 0.2% glycerin and incubated at 37° C for 7-10 days to reach exponential growth. Cultures were then pelleted (6000 rpm/15 min) and washed in a 0.264 M D-glucose solution as an osmoprotectant [13]. The washing procedure was repeated twice before adjusting turbidity to 1 of the McFarland scale.

### Viability assays for *M. smegmatis *and *M. tuberculosis *in the presence of DODAB dispersions

Cell viability was determined after incubation of cells (5 × 10^6 ^CFU/mL) with DODAB dispersions over a range of concentrations (0-1 mM DODAB) for 1 h in the case of *M. smegmatis *or 7 days for *M. tuberculosis*. The positive control was performed at DODAB concentration equal to zero. After interaction between cells and DODAB, serial dilution (10^-1 ^to 10^-4^) of each tube using a solution of D-glucose 0.264 M yielded 500 CFU/mL from which 100 μL were taken and plated in duplicate on MHA plates, before incubation (72 h/30°C) for *M. smegmatis*. Plating for *M. tuberculosis *was made on Middlebrook 7H10 agar supplemented with OADC and glycerol plates incubated in 5% CO_2_, 10% humidity and 37°C for 3 - 4 weeks. CFU counting was then performed and plotted as % cell viability as a function of DODAB concentration.

### Determination of minimal bactericidal concentration (MBC) for RIF or DODAB against *Mycobacterium smegmatis *and *M. tuberculosis *in 0.264 D-glucose solution

The medium used for diluting cells was D-glucose 0.264 M. Usually, evaluation of combined action of antimicrobial drugs is performed from chequerboard experiments and presented as the standard fractional inhibitory concentration index (FICI). Unfortunately, the chequerboard experiment in the culture medium is not feasible for cationic DODAB, which cannot remain in dispersion at usual ionic strengths of the culture medium. DODAB dispersions flocculated at very low ionic strength (at and above 5 mM monovalent salt) and interacted with acidic components of the culture medium [3]. This explained the use of the isotonic D-glucose solution for MIC determination. Briefly, for *M. smegmatis *twofold serial dilutions of RIF, and DODAB BF were prepared at final volume of 1 mL per tube. The final concentration of the antimicrobial agents ranged from 512 to 1 μg/mL for RIF and from 512 to 1 μg/mL for DODAB BF. Each tube was inoculated with bacterial cell suspension to a final concentration of 10^4 ^cells per tube and the plates were incubated at 30°C for 72 h. An aliquot 0.1 mL was taken from each tube, diluted 1:10 and 1:100, and cell viability was determined by plating 0.1 mL of each dilution on MHA plates in duplicate and incubating for 72 h at 30°C. The MIC endpoint was the lowest drug concentration that killed ≥ 99.9% of the inoculum. For *M. tuberculosis *drugs were diluted in microplates in a solution of D-glucose in concentrations ranging from 1-0.0039 or 512-1 μg/mL for RIF and DODAB, respectively, in a volume of 100 μl per well. After drugs dilution, each well was inoculated with 100 μl of the standardized bacterial suspension (10^5 ^CFU/mL). The plates were incubated at 37°C, 5% CO_2 _and 10% humidity for 7 days. An aliquot was removed from each well diluted 1:10 and 1:100 dilution and 50 μl were plated on 7H10 ágar. The plates were then incubated at 37°C, 5% CO_2_, 10% humidity, 3-4 weeks. MBC was determined as the lowest drug concentration that killed ≥ 90% of the bacterial inoculum.

### Determination of fractional inhibitory concentration index (FICI) for combinations of DODAB and RIF dispersions against *M. smegmatis *and *M. tuberculosis*

Twofold serial dilutions of RIF solutions or DODAB dispersions were distributed in tubes, in order to obtain final concentrations ranging from 512 to 1 μg/mL RIF or DODAB after interaction with cells. Thereafter, RIF diluted solutions were added of DODAB BF dispersion to yield a final DODAB concentration of 2 μg/mL after interaction with cells. Similarly the DODAB BF diluted dispersions were added of RIF solution to yield a final RIF concentration of 2 μg/mL after interaction with cells. Drug-lipid mixtures were incubated together for 1 h before adding the cell suspension. Each tube was inoculated with *M. smegmatis *cell suspension to yield 10^4 ^cells/tube and incubated at 30°C for 72 h. Thereafter, an aliquot of 0.1 mL was diluted 1:10 and 1:100, and cell viability was determined by plating and incubation for 72 h, 30°C. The lowest drug concentration that killed ≥ 99.9% of the inoculum was used to determine the fractional inhibitory concentration (FIC), defined as the ratio of the MBC of a drug used in combination with the MBC of the drug tested alone. The FIC index (FICI) was calculated as the sum of the FICs for the most equally effective concentration of drugs. According to Odds, FICI ≤ 0.5 corresponds to drug synergism, whereas an index > 4.0 represents antagonism [14]. Each experiment was carried out in duplicate and no variation was obtained among them. The microplate was used for the test of synergism between DODAB/BF and RIF against *M. tuberculosis*. A serial dilution of RIF was performed to yield final concentrations over the 1-0.0039 μg/mL range in 50 μL per well. Each well containing RIF dilutions was added of 50 μL of a 4 μg/mL DODAB dispersion. Furthermore, serial dilutions of DODAB BF (512-1 μg/mL) in 50 μL per well were added of RIF at 0.0078 μg/mL (a concentration value lower than MBC). RIF and DODAB BF were mixed and allowed to interact for 1 hour before addition of bacterial cells. In each tube 100 μL of the standardized bacterial suspension (10^5 ^CFU/mL) was added. The microplates were then incubated (37°C, 5% CO_2_, 10% humidity,7 days). An aliquot of each well was diluted 1:10 and 1:100, 50 μL of the final dilution was plated on 7H10 agar and incubated (37°C, 5% CO_2_, 10% humidity, 3-4 weeks). MBC was determined as the lowest drug concentration that killed ≥ 90% of the bacterial inoculum.

## Results

### Physico-chemical characterization of drug/DODAB assemblies

**Figure 2:**
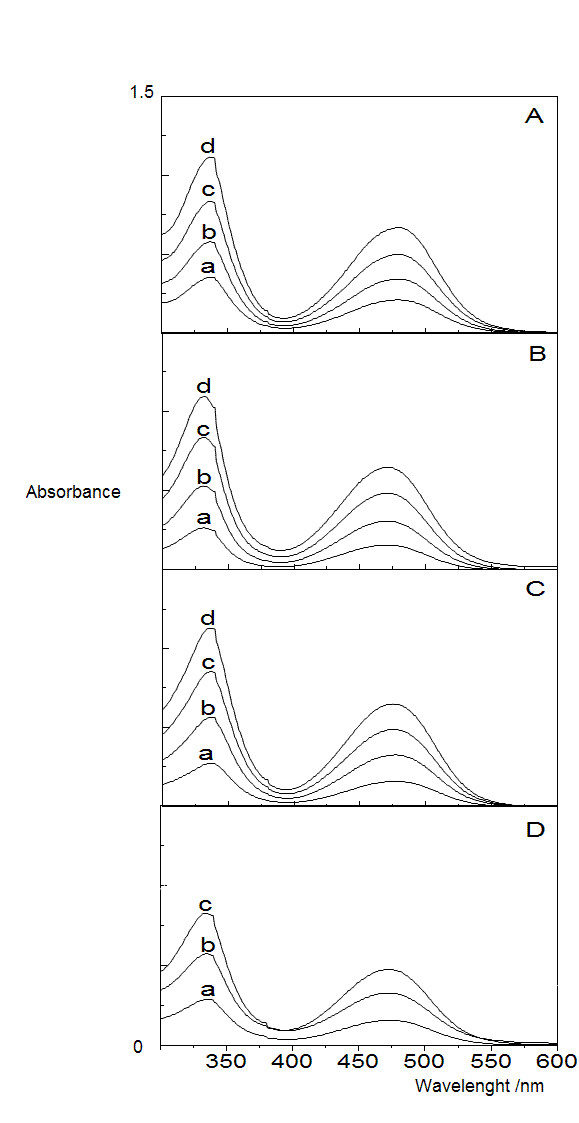
**Solubilization of RIF aggregates in DODAB bilayer fragments or LV**. Light absorption spectra for RIF in: DMSO (A), water (B), DODAB BF at 5 mM DODAB (C) and DODAB LV at 1 mM DODAB (D). RIF final concentrations were 16 (a), 32 (b), 48 (c) and 64 μg/ml (d).

The solubility limit for RIF in water is 1.3 mg/mL or 1.6 mM. Above this limit the drug aggregates in water solution. Optical spectra for the drug in different media such as water, DODAB BF or DODAB LV were similar to those for RIF in its best organic solvent, DMSO (Figure 2). Absorption maxima for the drug in water occurred at smaller wavelengths than those for RIF in its best organic solvent DMSO or in the DODAB dispersions (Table 1). In the presence of DODAB bilayers, the drug displayed absorption maxima at wavelengths practically equal to the one in its best organic solvent DMSO. The molar absorptivity (ε) of the drug in DMSO was 15,130 M^-1^cm^-1^. Similar values occurred in DODAB dispersions (Table 1).

**Table 1:** The DODAB bilayer as a solubiliser for RIF.

Solubilization medium	**λ**_**1max**_**/nm**	**λ**_**2max**_**/nm**	**ε at λ**_**1 max**_**/M**^**-1**^**cm**^**-1**^
DMSO	336	477	15,130
Water	331	468	13,890
DODAB BF	336	476	14,840
DODAB LV	334	476	15,000

Absorption maxima and molar absorptivities (**ε**) for RIF in different media.

**Figure 3:**
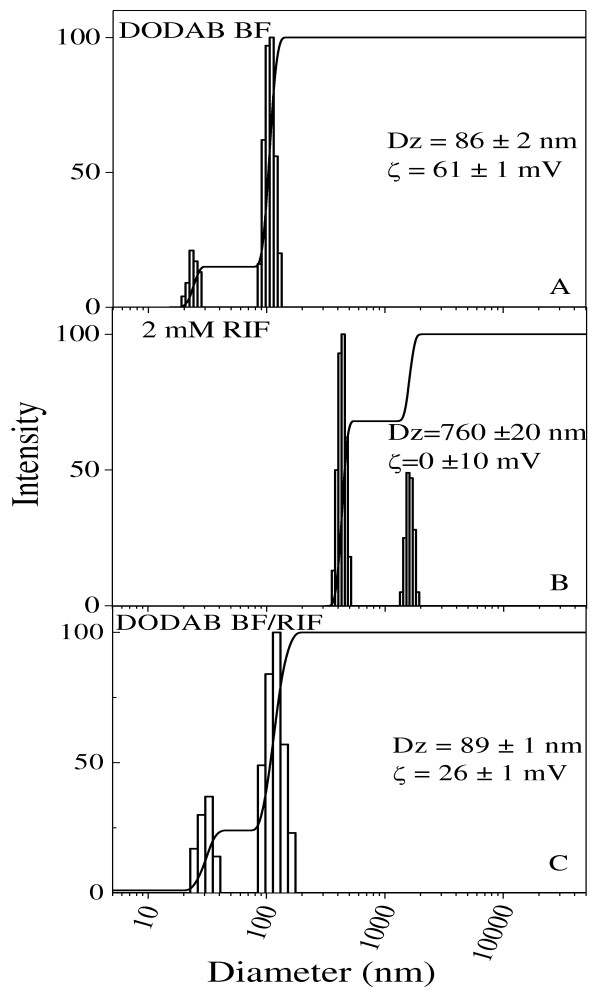
**Solubilization of RIF aggregates in DODAB bilayer fragments**. Size distributions for DODAB BF in water at 0.5 mM DODAB (A), RIF dispersion in water at 2 mM RIF (B) and mixture of both at the same final concentrations (C). Zeta-average diameter (Dz) and zeta-potential (ζ) are quoted in each subfigure.

Figure 3 showed the size distribution for DODAB BF at 0.5 mM DODAB in water (Figure 3A), 2 mM RIF in water (Figure 3B), and 2 mM RIF in DODAB BF (0.5 mM DODAB) (Figure 3C). Mean particle size for drug dispersion at 2 mM RIF was 760 ± 20 nm whereas the zeta-potential was 0 mV ± 10. When solubilized in the bilayer fragment, Dz was 89 ± 1 nm and zeta potential, 26 ± 1 mV (Figure 3C). This size was the same obtained for DODAB BF in the absence of drug 86 ± 2 nm. The zeta-potential of (26 ± 1 mV) was lower than the one in absence of drug (61 ± 1 mV) (Figure 3C).

**Figure 4:**
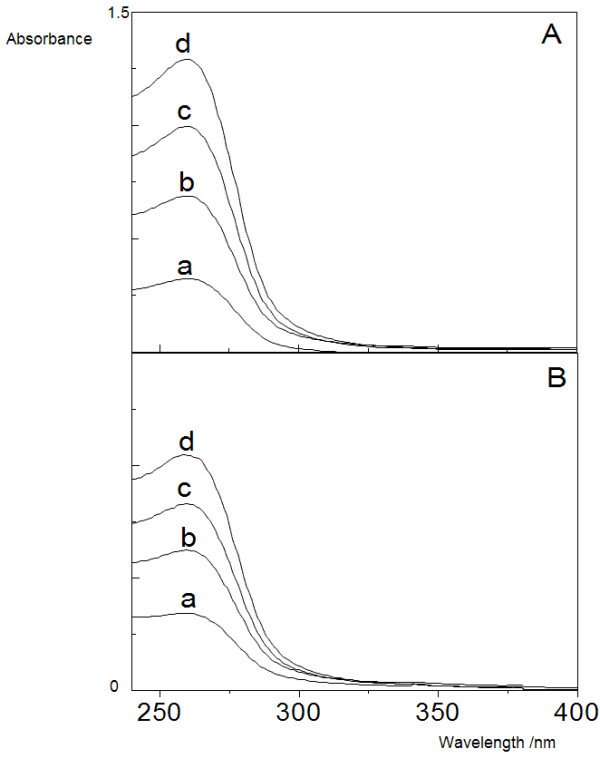
**Isoniazid solubility in water**. UV-VIS spectra of isoniazid in: water (A), DODAB LV at 2 mM DODAB (B). ISO final concentrations were: 10 (a), 20 (b), 30 (c) and 40 μg/ml (d).

Optical spectra for ISO in water were not affected by DODAB LV or BF (Figure 4). Its single absorption peak around 260 nm in water remained unchanged in the presence of DODAB bilayers. The molar absorptivities in water or DODAB LV were also similar, 2,995 and 2,717 M^-1 ^cm^-1^, respectively, again suggesting that ISO was not incorporated in the DODAB bilayer.

Table 2 showed absorbance values at 334 nm before and after dialysis in an experiment designed to determine RIF incorporation in DODAB dispersions. The control showed that RIF permeated freely through the dialysis membrane. For DODAB LV/RIF, the majority of drug molecules were retained inside the dialysis bag yielding 81% incorporation and ENT of 405 L/mol (Table 2). For DODAB BF/RIF, entrapment efficiency was 375 L/mol, a value slightly smaller than the one determined for DODAB LV/RIF. This might have been due to the extra amount of drug inside the vesicle aqueous compartment. By means of an alternative technique such as ultracentrifugation, 77.3% incorporation in LV at 2 mM DODAB was obtained. Total RIF concentration before ultracentrifugation was 0.075 mM. Thereafter only 0.017 mM remained in the supernatant. The difference between these two concentrations allowed the calculation of RIF incorporated in the DODAB LV dispersion as 77.3% in fair agreement with incorporation determined from dialysis.

**Table 2:** Eletrostatic attraction and hydrophobic effect massively drove RIF to DODAB bilayers

Sample	**A**_**before**_	**A **_**after**_	% Inc	ENT(L/mol)
RIF 0.1 mM	1.98 ± 0.05	0.12 ± 0.05		
DODAB LV 2 mM/RIF 0.1 mM	1.99 ± 0.05	1.62 ± 0.05	81	405
RIF 0.1 mM	2.30 ± 0.05	0.21 ± 0.05		
DODAB BF 2 mM/RIF 0.1 mM	2.30 ± 0.05	1.75 ± 0.05	75	375

Rifampicin incorporation in DODAB dispersions from RIF absorbance at 334 nm (A) before and after dialysis.

Dialysis experiments designed to determine ISO incorporation in DODAB dispersions revealed the leaky character of the DODAB LV towards ISO. After dialysis overnight ISO incorporation fell to zero (Table3). Other liposomes prepared from asolecithin or DODAB/DPPC 1:1 also yielded very low incorporation percentiles: 0% ISO incorporation in DODAB/DPPC large vesicles or 0.01% incorporation in asolecithin multilamellar vesicles after 12-24 h dialysis.

**Table 3:** The leaky character of liposomal bilayers towards isoniazid from dialysis experiments.

Sample	**A**_**before**_	**A **_**after**_	% Inc
ISO (0.1 mM control)	0.542 ± 0.010	0.061 ± 0.010	-
DODAB LV/ISO 0.1 mM	0.540 ± 0.010	0.052 ± 0.010	0
DODAB/DPPC 1:1/ISO 0.1 mM	0.504 ± 0.010	0.058 ± 0.010	0
ISO (10.0 mM control)	20.00 ± 0.010	0.017 ± 0.010	-

ASO MLV/ISO 10 mM	19.50 ± 0.010	0.400 ± 0.010	0.01

Isoniazid incorporation in DODAB, DODAB/DPPC 1:1 or asolecithin (ASO) liposomal dispersions from ISO absorbance at 260 nm (A) before and after dialysis. Final lipid concentration was 2.0 mM.

### Micobactericidal activity of DODAB alone or combined with rifampicin

**Figure 5:**
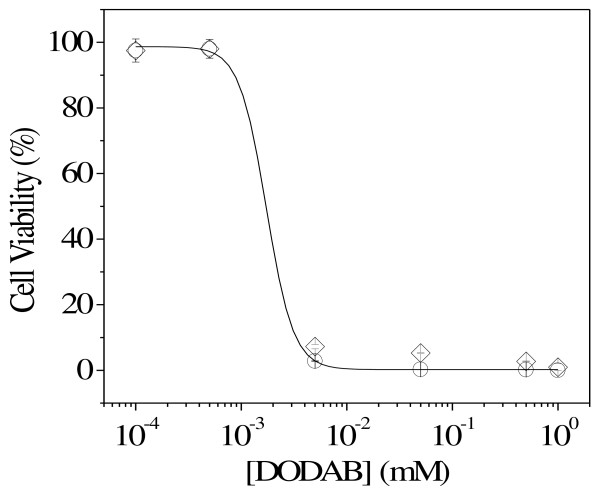
**Microbicidal effect of DODAB bilayers on *M. smegmatis***. Cell viability (%) for *M. smegmatis *at 3.5 × 10^6 ^CFU/mL as a function of DODAB concentration after 1 h of interaction time with DODAB LV (circles) or BF (diamonds).

**Figure 6:**
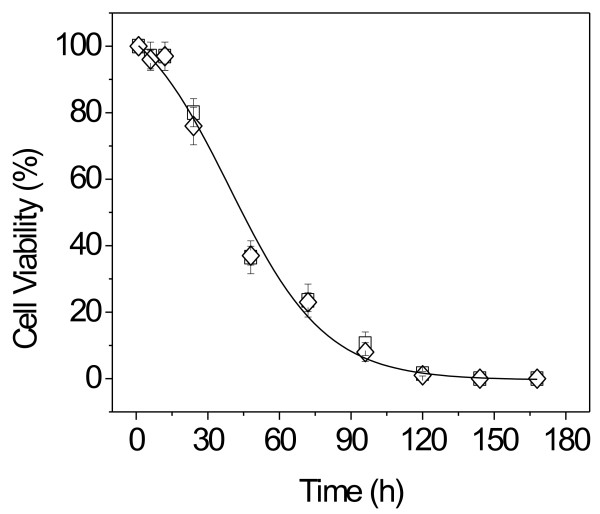
**Microbicidal effect of DODAB bilayers on *M. tuberculosis***. Cell viability (%) for *M. tuberculosis *at 1.8 × 10^6 ^CFU/mL and 0.1 mM DODAB as a function of interaction time with DODAB LV (diamonds) or BF (squares).

The effect of DODAB concentration for DODAB dispersed as DODAB BF or LV on *M. smegmatis *at 3.5 × 10^6 ^CFU/mL after 1 h interaction time is shown in Figure 5. Micromolar DODAB concentrations were effective against *M. smegmatis*. DODAB dispersed as LV or BF affected the cells similarly. At 0.002 mM DODAB, cell viability was 50%. After 120 h, 0.1 mM DODAB completely killed *M. tuberculosis *(Figure 6).

Table4 showed MBC for RIF alone or in combination with DODAB. The formulation DODAB/RIF yielded ΣFIC = 0.5 evidencing synergism between DODAB and RIF action against *M. smegmatis*. Against *M. tuberculosis*, ΣFIC = 1 suggested independent action for DODAB and RIF in the combination.

**Table 4:** DODAB and DODAB/RIF killed *Mycobacterium*.

	MBC (μg/mL)		FIC*	ΣFIC**
			
Microrganism	Drug	Combination		
	32 (RIF)	2 (2 DODAB BF)	0.0625	
*M. smegmatis*	4 (DODAB BF)	2 (2 RIF)	0.5	0.5
	4 (DODAB LV)	2 (1 RIF)	0.5	
	0.015 (RIF)	0.007 (4 DODAB)	0.5	
*M. tuberculosis*	8 (DODAB BF)	4 (0.007 RIF)	0.5	1
	8 (DODAB LV)	4 (0.007 RIF)	0.5	

Micobactericidal activity of RIF alone or in combination with DODAB.

## Discussion

RIF was partially anionic (~ 40%) at pH 7.4 and electrostatically interacted with the DODAB bilayer. The insertion of RIF in the DODAB bilayer was supported by zeta-potential decrease and could be understood from the favourable electrostatic attraction between the partially anionic RIF and the cationic bilayer which subsequently drove the hydrophobic effect. This was consistent with literature for other lipid bilayers loaded with RIF and ofloxacin [15] or for RIF interactions with neutral dimyristoil-L-α-phosphatidylcholine (DMPC) liposomes [16]. Together eletrostatics and hydrophobic effect solubilized RIF in the DODAB bilayer yielding the very large entrapment efficiency determined both for DODAB BF or DODAB LV (Table 2). In fact, incorporation of RIF was reported in various liposomal formulations such as dispersions of phosphatidylcholine (PC); dipalmitoyloglycero-PC (DPPC), distearoylglycero-PC (DSPC) with or without cholesterol [17]. RIF incorporation was evaluated in DPPC, DSPC or PC liposomes and reported at highest in DPPC and DSPC bilayers [18]. Similarly to DPPC and DSPC, DODAB also bears saturated hydrocarbon chains, yielding optimal and high RIF incorporation (Table 2). On the other hand, dialysis experiments showed that ISO permeated freely through the DODAB LV bilayer. This high permeability was also observed for vesicles composed of other lipids such as DODAB/DPPC 1:1 or asolecithin (ASO) multilamellar vesicles, though the multilamellar assemblies yielded percentiles of incorporation slightly above zero (Table 3). ISO solubility in water and permeability through the DODAB bilayer hampered ISO incorporation in DODAB BF or DODAB LV. Sustained release of ISO from DPPC multilamellar liposomes was observed over 24 h [19]. This reconfirmed the high permeability of this drug across phospholipid bilayers. DODAB LV did not retain ISO inside their aqueous compartment after the12-24 h dialysis procedure.

The slightly smaller RIF entrapment efficiency for DODAB BF in comparison to the one determined for DODAB LV (Table 2) might have been due to the extra amount of drug inside LV aqueous compartment which was not available for the open DODAB BF. Reproducibility of entrapment experiments was checked by ultracentrifugation and results were shown to be very reproducible.

Other studies have shown that the covalent combination of drugs with hydrophobic moieties restored or increased drug activity. Neomycin B had low activity against strains of methicillin resistant *S. aureus *(MRSA) and *P. aeruginosa*, whereas kanamycin A had low activity against MRSA and methicillin resistant *S. epidermidis *(MRSE) and *P. aeruginosa*. Recently, neomycin- or kanamycin A -cationic lipid conjugates were synthesized that displayed high activity against both MRSA and MRSE, respectively [20]. The conjugation of lipids increased the activity of both aminoglycosides against resistant strains: MIC for neomycin B against MRSA was 256 μg/mL. Its conjugate bound to lipid had a MIC of 8 μg/mL [20]. Our results showed that the noncovalent combination of RIF with the cationic lipid DODAB improved RIF antibacterial activity, reducing MBC from 32 to 2 μg/mL (Table 4). This work shows for the first time that drug-DODAB self-assembly results in enhanced micobactericidal activity at lower doses. These novel formulations may find useful applications in clinics. The use of low doses will possibly reduce drug toxicity *in vivo*.

## Conclusions

Electrostatic attraction and hydrophobic effect assembled RIF and DODAB bilayers yielding formulations with micobactericidal activity at reduced DODAB and RIF doses. DODAB by itself was also active against *Mycobacterium*. The present study *in vitro *is a necessary step before *in vivo *tests can be performed.

## List of abbreviations used

RIF: rifampicin; ISO: isoniazid; DODAB: Dioctadecyldimethylammonium bromide; BF: Bilayer fragments; LV: large unilamellar vesicles; Dz: Zeta-average diameter; ζ: Zeta-potential; MBC: Minimal bactericidal concentration; MIC: Minimal inhibitory concentration; TB: Tuberculosis; DPPC: dipalmitoylphosphatidylcholine; ASO: asolecithin; DMSO: dimethylsulfoxide; % Inc: Drug incorporation percentile; ENT: entrapment efficiency; MHA: Mueller-Hinton agar; FICI: fractional inhibitory concentration index; OADC: Oleic acid-albumin-dextrose complex; CFU: Colony forming unit.

## Competing interests

The authors declare that they have no competing interests.

## Authors' contributions

LB collected the data and helped to write the manuscript. EMM participated in the conception of the study and provided the *Mycobacterium *strains. AMC-R designed the study, interpreted the data and wrote the manuscript. All authors read and approved the final manuscript.

## References

[B1] World Health Organization The global tuberculosis control 2009 Geneve, Switzerland

[B2] Zager EM McNerney R Multidrug-resistant tuberculosis BMC Infect Dis 2008 8 10 10.1186/1471-2334-8-10 18221534 PMC2241599

[B3] Carmona-Ribeiro AM Bilayer-forming synthetic lipids: drugs or carriers? Curr Med Chem 2003 10 2425 2446 10.2174/0929867033456611 14529483

[B4] Carmona-Ribeiro AM Biomimetic particles in drug and vaccine delivery J Liposome Res 2007 17 165 172 10.1080/08982100701525530 18027236

[B5] Lincopan N Espíndola NM Vaz AJ da Costa MH Faquim-Mauro E Carmona-Ribeiro AM Novel immunoadjuvants based on cationic lipid: preparation, characterization and activity *in vivo* Vaccine 2009 27 5760 5771 10.1016/j.vaccine.2009.07.066 19664738

[B6] Carmona-Ribeiro AM Biomimetic nanoparticles: preparation, characterization and biomedical applications Int J Nanomedicine 2010 5 249 259 10.2147/IJN.S9035 20463941 PMC2865020

[B7] Carmona-Ribeiro AM Castuma CE Sesso A Schreier S Bilayer structure and stability in dihexadecyl phosphate dispersions J Phys Chem 1991 95 5361 5366 10.1021/j100166a080

[B8] Carmona-Ribeiro AM Synthetic amphiphile vesicles Chem Soc Rev 1992 21 209 214 10.1039/cs9922100209

[B9] Schales O Schales SS A simple and accurate method for the determination of chloride in biological fluids J Biol Chem 1941 140 897 884

[B10] Grabowski E Morrison I Dahneke B Measurements of suspended particles by quasi-elastic light scattering 1983 Wiley-Interscience: New York 199 236

[B11] Sobral CNC Soto MA Carmona-Ribeiro AM Characterization of DODAB/DPPC vesicles Chem Phys Lipids 2008 152 38 45 10.1016/j.chemphyslip.2007.12.004 18258186

[B12] Kagawa Y Racker E Partial resolution of the enzymes catalyzing oxidative phosphorylation: IX. Reconstruction of oligomicin sensitive adenosine triphosphatase J Biol Chem 1966 241 2467 2474 4223641

[B13] Helmerhorst EJ Reijnders IM van't Hof W Veerman EC Nieuw Amerongen AV A critical comparison of the hemolytic and fungicidal activities of cationic antimicrobial peptides FEBS Lett 1999 449 105 110 10.1016/S0014-5793(99)00411-1 10338113

[B14] Odds FC Synergy, antagonism and what the chequerboard puts between them J Antimicrob Chemother 2003 52 1 10.1093/jac/dkg301 12805255

[B15] Diekema DJ Pfaller MA Jones RN Doern GV Kugler KC Beach ML Sader HS Bermudez M Martinez E Mora M Sarigras ML Madariaga MA Molecular and physicochemical aspects of the interactions of the tuberculostatics ofloxacin and rifampicin with liposomal bilayers: a 31P-NMR and DSC study Colloids Surf A: Physicochem Eng Aspects 1999 158 59 66 10.1016/S0927-7757(99)00131-4

[B16] Rodrigues C Gameiro P Pietro M de Castro B Interaction of rifampicin and isoniazid with large unilamellar liposomes: spectroscopic location studies Biochim Biophys Acta 2003 1620 151 159 12595084 10.1016/s0304-4165(02)00528-7

[B17] Zaru M Mourtas S Klepetsanis P Fadda AM Antimisiaris SG Liposomes for drug delivery to the lungs by nebulization Eur J Pharm Biopharm 2007 67 655 666 10.1016/j.ejpb.2007.04.005 17540552

[B18] Gursoy A Kut E Ozkirimli S Co-encapsulation of isoniazid and rifampicin in liposomes and characterization of liposomes by derivative spectroscopy Int J Pharm 2004 271 115 123 10.1016/j.ijpharm.2003.10.033 15129978

[B19] Chimote G Banerjee R *In vitro *evaluation of inhalable isoniazid-loaded surfactant liposomes as an adjunct therapy in pulmonary tuberculosis J Biomed Materials Res B: Appl Biomat 2010 94B 1 10 10.1002/jbm.b.31608 20524179

[B20] Bera S Zhanel GG Schweizer F Design, synthesis, and antibacterial activities of neomycin-lipid conjugates: polycationic lipids with potent gram-positive activity J Med Chem 2008 51 6160 6164 10.1021/jm800345u 18778047

